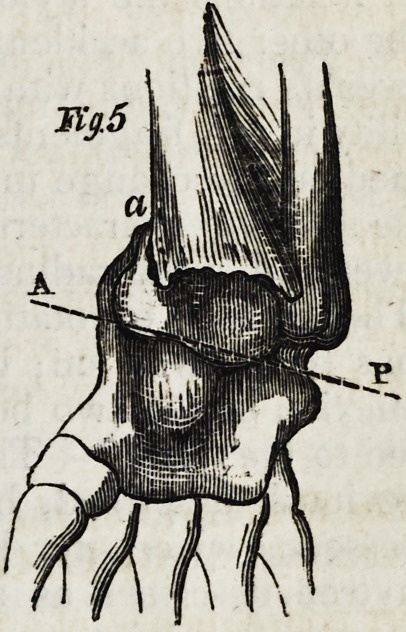# Surgery

**Published:** 1836-07

**Authors:** 


### SURGERY.

### *On the Treatment of Inflammation of the Testicle by means of Compression.*

By J. C. F. FRICKE, Surgeon to the General Hospital in Hamburg.

[IN presenting to our readers the following important document relative to a new mode of treating Hernia humoralis, or swelled testicle, we shall for the most part make use of the author's own words, merely omitting phrases and brief paragraphs, here and there, which do not seem essential. It may be well to inform the reader, that Dr. Fricke is a surgeon of great reputation and of most extensive experience, and the author of some surgical works of much practical value.]

I had long meditated (says Dr. Fricke,) on the discovery of some means to obviate the tediousness and other numerous inconveniences attending the common mode of treating inflammatory affections of the testicle, by leeches, poultices, &c.; and at length it occurred to me that *compression,* which I had found so very serviceable in some analogous cases, offered the fairest prospect of a favorable result. The event completely answered my expectations; and I had soon the pleasure to perceive how, by means of this, the disease could be removed, in a simple, easy, and surprisingly rapid way.

Generally speaking, compression may be employed in every kind of inflammatory enlargement of the testicle, and from whatever cause produced. We have found it equally useful in cases arising from gonorrhœa, whether springing from sympathy in the inflammatory stage, or originating in what is called suppressed claps, and in such as have arisen from external injuries. The degree or period of the inflammation makes no difference.

The only contra-indication to the employment of this treatment, worthy of consideration, has been found in an affection of the general system. For instance, if the local inflammation had arisen from errors in diet, such as abuse of spirituous liquors, or if, contemporaneously with it, considerable disorder of the gastric system had shown itself, it was found necessary to remove this state before recourse was had to compression; as, otherwise, the usual result was not obtained, and the employment of compression was obliged to be postponed for a period.

In many cases the compression at first increased, in some degree, the pain of the inflamed testicle; in some cases (particularly when applied too forcibly,) it produced great pain; but this never continued long: the patient, after a short time, often in a quarter of an hour, and even in cases where the pain had been extremely severe, finding himself so completely relieved as to be able to leave his bed and to walk about in his room.

In inflammatory swellings of quite recent origin, a single application of the compression was found sufficient, in many cases, to remove the disease. When it was of longer duration, (say, from three to eight days,) it was found necessary to repeat the compression two or three times. Swelling of the spermatic cord, if it was not very considerable, did not at all contra-indicate compression; nor yet did other contemporaneous local affections, such as buboes, ulcers, &c. When a general febrile state was produced by the orchitis, compression was found the best means speedily to remove it, at least where the vascular reaction was not too great; although, in extremely rare cases, this was produced by the compression itself.

The unpleasant part of the treatment by compression was, as I have said, its occasioning pain in some cases. This result was observed chiefly in the early period of my practice, and I considered it as owing to our making the compression too strong. In my latter practice, on avoiding this, we heard no more of pain being produced by it. In some cases, in which the affection had been previously treated by cataplasms, &c., and where we had only made one application of the compression, there still remained for some time a slight painful swelling of the testicle; but it gradually disappeared.

In several cases I had occasion to observe, as the consequence of compression, nausea, inclination to vomit, and bitter taste in the mouth, coming on without any other evident mark of gastric disorder. When this was the case, compression evidently was of no avail; the pain remitted little or not at all, and the swelling did not decrease. On removing the compression, giving an emetic, or applying a poultice to the stomach, the symptoms of disturbance soon disappeared. In the few instances in which this affection of the stomach was observed, the compression had been for the most part too strong; in two of them, however, it seemed to depend on previous disease in the abdomen. It is however to be observed, that the cases in which this sympathy exists in such a degree as to give occasion to gastric disorder are, generally speaking, so rare as not to be regarded as any drawback on the superiority of this mode of treatment. It is necessary, however, in all cases where such a disposition shows itself, immediately to put an end to the compression.

The good effects of the compression show themselves very soon after its application, and the speedy abatement of the pain is always the surest sign of its efficacy. If the pain continues some hours in any considerable degree, a general disorder of the system may be looked to as explaining the failure of the treatment.

I will now give a comparative statement of the results of the treatment of orchitis by leeches, cataplasms, &c., and of that by compression, taken from the journals of the General Hospital, since the commencement of the practice in 1832. In all, we have compared seventy-four cases: of this number, fifty-one may be regarded as acute cases, or cases in which the symptoms of inflammation were strongly marked, and twenty-three as chronic cases, or cases in which the inflammatory symptoms had more or less remitted. Of the first division (of fifty-one), eighteen were treated with leeches, cataplasms, &c., and thirty-three by compression;—of the second division (of twenty-three), nine were treated with poultices, leeches, &c., and fourteen by compression. The following are the results of the two different kinds of treatment, as regards the time occupied during the case:—Of the thirty-three cases of acute orchitis treated by compression, the average period of treatment was nine days;—of the eighteen acute cases treated without compression, the average was thirteen days:—of the fourteen chronic cases where compression was employed, the average period of treatment was twelve days;—of the nine cases submitted to other treatment, the average was fourteen days. Such were the average results; some of the comparative results of the two kinds of treatment, in reference to individual cases, were as follows:—Of the thirty-three acute cases treated by compression, five were cured in three days; five, in five or six days; six, in seven days;—of the eighteen acute cases treated by other means, one case was cured in three days; one, in five days; two, in from seven to eight days, seven, in from eight to eleven days. In regard to the chronic cases, out of the fourteen treated by compression, one was cured in two days, and the greater number in ten or twelve days; while, of the nine cases in which cataplasms, leeches, &c. were used, the cure took place in no case in less than eight days.

Latterly, when experience had enabled me to treat the disease with more circumspection, the results of compression was much more favorable. In the present summer (1835,) I treated in this way seventeen cases, which are not included in the above statement. Of these were cured in one day, one; in two days, four; in three days, four; in four days, two; in five days, three; in nine days, one; and two in ten days. The three last were severe and unfavorable cases. In nearly two thirds of the whole of the above-mentioned cases, no hardness or swelling of the testicle remained behind.

I will now describe the manner in which I apply compression. At first I attempted to compress the testicle against the thigh and pelvis, by passing over it long and wide strips of sticking plaster, from the nates up to the abdomen. I was soon forced to give up this plan, as well because the compression produced by it was neither secure nor equal, and the patient was forced to keep himself in bed, and, even while there, to avoid all considerable movements. After many other unsuccessful attempts by means of temporary bandages, &c., I at length adopted the following, which is proved by experience to be the best.

For the purpose of compression, I employ strips of sticking plaster; the plaster being made very adhesive, but not of too irritating materials,* and spread on linen the breadth of the thumb. No preparatory measures are required; no leeches, cataplasms, &c.

In slighter cases the patient may stand before the surgeon, leaning against the wall, or he may rest on the edge of the bed or sofa, in such wise that the scrotum may hang freely down. If the scrotum and neighbouring parts are much covered with hair, this must be removed; but, generally speaking, this is unnecessary.

The surgeon takes the scrotum in one hand, and separates the diseased from the sound testicle, while with the other hand he gently stretches the skin of the scrotum over the former; the spermatic cord is isolated in the same manner. If the testicle is much swollen, it must be held by an assistant; otherwise, it suffices for the patient himself to keep the sound testicle somewhat separate from the diseased. The surgeon now applies the first strip of plaster over the isolated spermatic cord, about a finger's breadth above the testicle, holding the end of the strip with his thumb, and passing it round the cord. He proceeds in the same way with the second strip, which must either in part or altogether cover the former. The first part of the process must be carefully done; the strips must compress the cord closely, (and for this purpose it must be kept approximated to the skin, which is to be tightly stretched over it;) otherwise, when the other extremity of the testicle is compressed, the upper end will be apt to slip upwards through the loose rings of sticking plaster; a circumstance not only occasioning pain, but rendering the whole operation abortive. In this manner we proceed, laying strip after strip, the last always lying over the former by a third of its width, until we have reached the thickest part of the testicle, and where it begins rapidly to decrease in diameter. The surgeon now changes his mode of proceeding, and, laying hold of the testicle already covered, passes his strips from above downwards over the lower portion of the testicle, and up over the back part. In this way the whole remaining portion of the testicle is closely enveloped and compressed. I have already said that the compression must not be too great; and in most cases the surgeon will be able to judge as to the proper degree by the speedy disappearance of all the pain which had previously existed.

If both testicles are affected, we proceed to envelope one in the manner now described: when this is done, it will be found that there is not room left for applying the circular strips in the same manner to the other; we are therefore under

* The following composition, contained in our Codex, is the plaster which I have employed for some years, and has been found preferable to all others:

℞. Emplastri Lithargyri, partes sex;
Colophonii pulverati (Picis nigræ,) partem unam. Seorsim liquata commisceantur.

The Emplastrum Lithargyri employed is made as follows:

℞. Lithargyri subtilissime lævigati, lib. v.
Olei Oliv. lib. ix. M. Coque igne moderato, spatula lignea semper agitando et pauxillum aquæ subinde instillando, donec Lithargyrum perfecte solutum sit, &c.

the necessity of including both testicles in the circular strapping, the testicle already covered serving as a point of support for the other. Over the lower portion of this second testicle the strips are passed, as in the former case, from behind backwards.

In some cases where the skin is irritable, ulcerations take place: in this case small slits must be cut in the plaster, and a goulard lotion applied, which soon heals them.

Generally speaking, the patients can leave the bed immediately after the strapping, and walk about the room; and, in cases where the inflammation is not very great, or has been taken early, they may even go out and work a little.

The renewal of the straps must depend on the decrease of the swelling and other symptoms. In many cases one application suffices; otherwise, we remove the plasters when they have become so loose as to admit the introduction of the scissors between them and the skin.

Any other treatment the patient may require must depend on the complications of the disease: the orchitis, as such, needs nothing besides the compression.

In those inflammations of the testicle which originate from blows or pressure, &c. compression has proved the best treatment. Here, if the inflammation ran very high, I have usually applied leeches in the first instance, and kept on poultices for one or two days; but in slighter cases I had recourse immediately to compression.

The following are the principal advantages which the treatment of orchitis by compression possesses over other methods:

1. The speedy removal of pain.
2. The quick removal of the disease itself.
3. The simplicity of the method, and the slight trouble thereby given to the patient.
4. The small expense of the treatment.
5. The comparatively slight care and attendance required on the part of the surgeon.—The two last points are of considerable importance in hospital practice.

*Zeitschrift für die gesammte Medicin.* B. i. h. 1. 1836. *Hamburg.*

---

### *On Fractures of the Inferior Extremity of the Radius.*
By G. Goyraud, M.D., of Aix.

A FREQUENT consequence of a fall on the hand is a painful swelling of the wrist, hand, and lower extremity of the forearm. This tumefaction is accompanied by a deformity consisting in an unnatural projection of the lower extremity of the ulna; a change of form in the forearm, which is rounded inferiorly; inclination of the wrist outwards and generally backwards, and of the hand in the contrary direction. Generally the only treatment consists in emollient applications. The swelling slowly declines, and the motions are not free for a long period. Six months after the fall, the wrist-joint has not recovered its suppleness. As the swelling disappears, the projection of the lower end of the ulna is more apparent: inequalities are felt on the palmar surface of the inferior extremity of the radius. Eventually the joints regain their mobility, but the deformity remains throughout life. (Figs. 1 and 2.)

This accident has been considered by some to be a diastasis of the inferior radioulnar articulation, by others as a sprain; by Petit and Boyer as a dislocation of the wrist: but no external violence could separate from each other the lower ends of the radius and ulna; no sprain could change the direction of the hand; and, if the possibility of luxations of the wrist are admitted, (which Dupuytren doubts,) still they must be very rare, whilst this accident is very common. It can only be accounted for by a fracture of the radius.

Fig 3

Fractures of the carpal extremities of the radius are generally oblique from above downwards, and from the dorsal to the palmar surface. (Fig. 3, line F R.) Out of forty-seven instances of fractured radius, forty-three were in this direction; two others were oblique fractures from above downwards, and from the palmar to the dorsal surface; in one other the inferior fragment was fractured vertically, and in another there was a star-like fracture into many pieces. In the most common variety, the obliquity was of various degrees, sometimes nearly transverse. (Fig. 4, line F R.) In the common oblique fracture the inferior fragment is forced, by the violence of the blow, and the action of the muscles passing from the forearm to the hand, from below upwards and from before backwards. (Fig. 3.) The superior fragment is drawn towards the interosseous space by the action of the

Fig. 4.

Fig. 5

two pronator muscles. (Fig. 5.) The consequence of this displacement is a diminution of the breadth of the inferior part of the forearm and interosseous space, (fig. 5;) a depression on the external side of the radius, some lines above the wrist, (fig. 5, *a*;) an inclination of the carpal articulatory surface of the radius outwards (fig. 5, line A P,) and backwards (fig. 3, line A P.) Cline and Cooper have attributed the prominence formed anteriorly above the wrist to the displacement of the inferior extremity of the *upper* fragment by the pronator quadratus; but M. Goyraud is convinced, from numerous dissections, that the *inferior* fragment is displaced, producing the prominence. The more oblique the fracture, the greater the displacement: in transverse fractures the violence only causes the displacement, and this may be so great as to simulate luxation of the wrist, (fig. 4;) an error which may be strengthened by the fact, that, after reduction, there is no tendency in the parts to become again displaced. The carpus follows the direction of the articular surface of the radius, so that the articulation of the wrist takes a direction separating it from the inferior extremity of the ulna, which consequently forms a projection remarked by Petit and Boyer, who believed it to be a consequence of luxation of the wrist. The hand would follow the same direction, if it were not for the internal lateral ligament of the joint, which prevents the hand turning outward; the flexor muscles, rendered tense by the wrist being thrown backwards in the common oblique fracture from behind forward, draw the hand forwards; in the fracture in the opposite direction, the extensor muscles draw the hand backwards. In the great majority of cases, the hand is fixed in the state of adduction and a little inclined forwards; sometimes, but rarely, backwards. If the violence has ruptured the lateral ligaments, or separated the styloid process of the ulna, the hand and wrist are in a state of adduction. (Fig. 1.)

These oblique fractures of the radius are extremely frequent: they generally are caused by falls on the palm of the hand, but sometimes on the dorsal surface, the hand being strongly bent forwards. The indications of this fracture are—an unnatural projection of the lower end of the ulna, a depression on the radial border of the forearm some lines above the wrist-joint, a little increase in the dorso-palmar diameter, and a little diminution in the radio-ulnar diameter of the forearm, at a point corresponding to the depression on the radial edge; pain in the lower extremity of the *radius*, augmented by pressure at this point, but not by the motions of the joint; pain also beneath the lower end of the ulna, from the dragging or rupture of the internal lateral ligament. In the commonest oblique fracture from above downwards and behind forwards, the wrist is inclined backwards, its axis forming an angle, more or less marked, with the forearm. (Fig. 2, lines A B and B M.) From thence a depression on the dorsal face of the forearm, over the radius, and ten or twelve lines above the wrist, and a large prominence, convex from above downwards, on its palmar surface. (Fig. 2, *a*.) The hand is bent forwards; and this inclination is more considerable in proportion to the deviation of the wrist. In the rare fracture from above downwards and before backwards, the wrist is bent forwards and the hand backwards. The inequalities are felt before the swelling begins, and when it is partly dissipated. Transverse fractures with great violence, and separation of the epiphyses, may be mistaken for dislocations; but, as Dessault remarked, in luxations the styloid process of the radius loses its relation to the carpus; in the other two accidents it is no longer in the same line as the radius, but it preserves its relations with the hand. Crepitation is often absent, from the want of mobility in the fragments and from the swelling. To sum up: the diagnosis is formed on the change in the direction of the axis of the wrist and that of the hand; the swelling characteristic of fractures; the pain seated not in the joint, but in the lower end of the radius, and the unusual projection of the lower end of the ulna. The absence of rotation in the end of the radius during pronation and supination has been dwelt on; but, from the large surfaces of the fracture and intimate connexion of the two bones, must not the motion of one be necessarily communicated to the other? This fracture is without danger: it leaves behind a deformity previously described, but M. G. has never known it produce obliteration of the interosseous space, nor loss of the motions of pronation and supination, which Dupuytren describes as a consequence of its being overlooked. Under such circumstances, the joint remains for a long time almost without motion.

*Treatment.* To reduce the fracture, the forearm must be bent and placed in a position between pronation and supination; an assistant produces counter-extension by seizing the lower part of the forearm, whilst another extends the limb by drawing the hand gradually outwards, and slightly inclining it towards the ulnar border of the forearm. The surgeon pushes the flesh of the two sides of the forearm into the interosseous space, and then puts the broken surfaces into apposition. The fracture is easily reduced, but retained with difficulty in its situation. The apparatus employed by M. Goyraud consists of two splints, about the breadth of the lower end of the forearm, one of which is from eighteen to twenty lines shorter than the other, and its inferior extremity cut off obliquely; two graduated interosseous compresses; and two pads, one between three and four inches long, and the same thickness as the middle of the graduated compress; the other an inch in length, and like a wedge, its base being about as thick as the anterior interosseous compress. The interosseous compresses are applied on the two faces of the forearm, parallel to the interosseous space, and descending to an inch above the joint; below this point they are replaced by the pads, the larger one over the dorsal aspect of the wrist, and the wedge-like pad on the palmar side, with its base next to the graduated compress, and its apex to the carpus. The longer splint, applied over the dorsal graduated compress and pad, is to descend to the posterior surface of the metacarpus; the shorter splint is placed over the palmar compresses and pad, its oblique extremity being inferior, and the acute angle of this extremity towards the radial edge of the limb, so that it is applied with the interposition of the cuneiform pad against the superior part of the prominence formed by the os pisiforme and os scaphoides. A tight roller confines the whole. The advantages of this method are the following:—As the interosseous space terminates an inch above the wrist-joint, the long graduated compresses generally used to prevent the bones coming in contact are of no use; but, by substituting pads whose surface corresponds (as these do) to the shape of the lower end of the radius, this bone is efficiently acted upon. The effect of the oblique extremity of the splint is to change the direction of the line formed by the prominence of the os pisiforme and scaphoid process, which is almost horizontal, into an oblique line running from above downwards and from the ulnar to the radial border of the limb; that is to say, to fix the hand in the state of adduction, and to oppose more certainly the reproduction of the displacement; an indication which Cline and Sir A. Cooper attempted by the weight of the hand, which they allowed to hang out of the sling. During the last two years, this treatment has been adopted by M. Goyraud in eleven cases with complete success.

[This active-minded and intelligent provincial surgeon temperately complains of his views of the nature of this frequent accident (which were first published in 1832,) being copied almost verbatim into the "Leçons Orales" of M. Dupuytren, as the original production of that eminent man. We should be unwilling to believe that Dupuytren, who in a clinical lecture might have thought it unnecessary to mention authorities, was a party to this printed plagiarism: indeed, the gross flattery of him by his reporters in almost every page renders it probable that the late Baron never revised the lectures.

We observe, in a subsequent number of the same Journal, a note of a *Reclamation* by Dr. Brière de Boismont, in which he asserts that Dupuytren's opinions respecting fractures of the radius were published in certain theses so far back as 1822,—viz. ten years before the publication of Goyraud. We are not able to decide on their respective claims.]

*Journal Hebdomadaire des Sciences Médicales, No.* 6, *Fevrier* 6, 1836.

---

*Surgical Observations.* By Professor CHELIUS, of Heidelberg.

[IN the first number of a new series of the Heidelberger Klinische Annalen, recently published under the name of *Medicinische Annalen,* this eminent surgeon has given an elaborate and highly interesting Report of his Surgical and Ophthalmological Clinical Practice in the Heidelberg Hospital, from 1830 to 1834 inclusive. We regret that our limits will only permit us to give a brief notice of some of the more important surgical observations contained in it.]

1. *Amputation.* Professor C. mentions that, out of twenty-nine cases of amputation, he lost only two patients. The circular incision was in every instance put in practice, and ligatures employed to secure the bleeding vessels; in no case torsion. Two alone required the removal of the dressings for after-hemorrhage; in all the rest they were allowed to remain untouched as long as possible; in some till the third week, at which period the wound was found perfectly closed.

2. *Lithotomy in the Female.* After discussing fully the merit of different plans that have been proposed, Professor C. adopts the method of incising perpendicularly downwards, (a modification of Bromfield's operation;) and for the following reasons: The urethra, throughout its whole extent, lies immediately upon the anterior wall of the vagina, as is likewise the case with the bladder. By the incision so directed these two points alone are implicated; the execution of the operation is simple, nor is it likely to give rise to any considerable loss of blood. The extraction of the stone will be attended with the least possible difficulty; and, should the large size of the stone demand it, the incision can be commodiously prolonged. Owing to the exact apposition of the vagina to the urethra and to the neck of the bladder, there exists a constant parallelism between the wound of the vagina, of the urethra, and of the cervix vesicæ; the urine finds a ready way of egress, and no danger need be apprehended from infiltration. From being able, in case of need, to carry the incision along the neck into the body of the bladder, every risk from pinching or tearing the cervix vesicæ is removed; lesions much more apt to produce subsequent incontinence of urine than the mere section of the neck.

As disadvantages resulting from this mode of operation, are enumerated the danger of vesico-vaginal fistula and permanent fissure of the urethra. The first of these objections must be opposed by the various arguments adduced in support of the vesico-vaginal section generally. In this respect the conditions here are much more favorable than in the instance of recto-vesical lithotomy; for here the vagina is empty, and the entrance of foreign matters into the cavity of the bladder, as fæces from the wounded rectum, rendered impossible. Nor is any inconvenience to be apprehended from the remote chance of the influx of fluid during the flow of the catamenia. The second objection is supported neither by anatomical nor physiological grounds, and is directly refuted by Professor C.'s own proper experience.

*Operation.* The grooved staff is introduced with its handle sustained vertically by the assistant, and its concavity pressed up against the pubic arch: in this manner the parts to be incised are more securely fixed, the entrance of the vagina somewhat widened, and the finding the groove on the staff facilitated, and, lastly, the section downwards of the urethra and vagina accomplished without trouble. The incision may be performed with a probe-pointed bistoury introduced to the requisite depth along the groove, and then made to cut its way outwards, dividing in its course the neck of the bladder, urethra, and anterior wall of the vagina, to the full extent wanted: or it may be executed by means of the lithotome caché of Frère Côme. Should the incision of the bladder be too small, it must be enlarged by means of the probe-pointed bistoury, conducted along the left index-finger.

Reports of three cases are related wherein the above plan answered.

3. *Scrotal Calculus.* This case is remarkable from the multitude of concretions removed, amounting to twenty-seven in number. The patient was fifty-five years of age, and attributed the origin of the complaint to a fall upon the perineum, about twenty years previously.

4. *Bronchocele, (Struma Lymphatica.)* Professor C. is of opinion that, in every case of this disease where the nutritious vessels are much enlarged and easily to be felt, their obliteration by means of ligature is equally indicated, as in the aneurismal form of the affection; for, although the diminution of the swelling in Struma lymphatica, after the supply of blood has been checked by tying the superior thyroid arteries, does not proceed with such rapidity, nor to the same degree, as in the vascular goitre, still however such a decrease in the bulk of the tumour will be obtained, that the inconveniences it had created will be in a great measure lessened or altogether removed.

The operation is of the simplest description. The rule given is to make the incision correspond, in direction and situation, with the course in which the arterial pulsations are most distinctly perceptible to the finger of the operator. This will most frequently be found to be between the omo-hyoïdeus muscle and the point at which the vessel is entering the gland; often, however, between its origin and the same muscle. An advantage attending this method is, that, if it should not fulfil the desired end, diminution of the morbid growth, other means can, with greater confidence, be put in practice.—Four cases are reported in which it proved of great service, and in no one was it productive of the least bad consequence.

5. *Erectile Tumours.* Creosote was tried as a topical application in several cases of this affection, and especially Nævus maternus in infants. The only effect its continued application seemed to produce was the formation of a superficial dry crust, which came away, leaving the tumour in statû quo: indeed, in one case, the volume of the nævus appeared to have augmented under the use of the creosote. From its inefficacy, he was obliged to have recourse to caustic, which he pronounces unfailing in its effects, and preferable to the knife, from the danger of mortal hemorrhage from the latter.

6. *Removal of an Abdominal Tumour.* This is another addition to the sepulchretum of operations in that cavity. The tumour was of a fibrous texture, of considerable magnitude, attached by a pedicle to the uterus. The patient never recovered the shock of the operation, which she outlived seventeen hours. It is justice to state, that its performance took place at the earnest solicitation of the patient, in opposition to the advice and opinion of her medical men.

7. *Teleaugiectasia Lipomatodes.* Under this strange name is detailed the history of a singular case of mixed tumour, partly erectile, partly lipomatous, occurring in the hand of a tailor, chiefly between the thumb and the metacarpal bone of the index-finger. As its presence interfered with the use of the needle, Professor C. determined to try the effect of tying the radial artery. Soon after the operation, so great was the amendment that he was again able to resume his handicraft. It is worthy of remark, that, in consequence of the supply of blood being diminished, it gradually lost its erectile character, assuming more and more that common to lipoma.

8. *Stricture of the Œsophagus.* For the permanent cure of stricture of the œsophagus, Professor C., taking advantage of the principle introduced by Ducamp for strictures of the urethra, employs an oval ivory dilatator, attached upon a common œsophagus bougie, about an inch and a half from its extremity. An ordinary œsophagus bougie is first inserted, to ascertain the existence and situation of the stricture. Should this fail to make a passage, a thinner bougie must be used. Where the coarctation is considerable, it is sometimes necessary to use middling-sized urethra bougies. The bougie is left in ten or fifteen minutes each day, gradually exchanging it for one of larger caliber, until the dilatator is permitted to enter, which patients are found to endure quite as well as the ordinary sounds. Under this plan there is rapid improvement; and, after the lapse of a few days, a second thicker dilatator may be substituted for the former, until perfect dilatation is effected, and deglutition rendered free. For some time after the cure it is advised to introduce the instrument once every five, eight, or fourteen days, to prevent relapse. All instruments for the cure of stricture are always to be introduced by the mouth.

*Heidelberg Medicinische Annalen,* Band. i. H. i. 1835.

---

### *On the Use of Belladonna as a Topical Application in Retention of Urine, Spasmodic Contractions of the Uterus, and in Strangulated Hernia.*

[THE well-known relaxant effects of belladonna on the iris, &c., has naturally led to its use in cases where spasm was known or assumed to exist in other parts. M. Guerin, of Bourdeaux, was the first, we believe, who employed it in spasmodic strictures of the urethra, in the form of ointment spread on a bougie; and he states that he found the same remedy, applied in the same manner, effectual in the case of strangulated hernia. Since then, belladonna has been frequently used topically in similar and analogous cases; and we shall here extract the heads of a few of the more recent which have met our eye in some of the foreign journals.]

### I. *Efficacy of Extract of Belladonna in Retention of Urine.* By M. GERARD, late chief Surgeon of the Hospital at Avignon.

CASE I. A lady, æt. 36, was delivered of her first child, after a long and severe labour, at one A.M., on the 16th November, 1834. Nine hours thereafter it was discovered that the urinary bladder was immensely distended and painful, no water having been passed since the commencement of the labour, and there being still an incapacity to do so. No attempt seems to have been then made to introduce the catheter; the surgeon contenting himself with ordering "vegetable lemonade, and an emollient poultice to the hypogastrium." At nine P.M., no urine having been passed, (now three days,) matters were of course worse, and then the surgeon seems for the first time to have thought of the catheter, but he could not succeed in its introduction, owing to what he terms "a manifest coarctation of the urethra." Being deterred by the patient's debility from the use of general and local bleedings or the warm bath, M. Gerard prescribed an ointment composed of two drachms of Extract. Bellad. to one ounce of lard, and ordered it to be rubbed on the hypogastrium and labia. The first friction was made at midnight, the next at three A.M.; and shortly after this last the patient began to make water in small quantities, with much pain. The frictions were continued through the day, and the urine at length flowed plentifully.

CASE II. A man, æt. 49, was attacked with retention of urine, accompanied with fever, for which he was copiously bled and leeched on two successive days, without relief. On the third day, frictions with the belladonna ointment were used on the hypogastrium and perineum. After the third friction there was a slight discharge of urine; and on the following day, the frictions being continued, the patient was completely relieved.

Case iii. A man, æt. 24, suffered very acute pains in the region of the bladder, attended with retention of urine for three days, the consequence of a severe blow. After the failure of general and local bleedings, and the warm bath continued for four hours, the belladonna ointment was had recourse to, and the urine flowed after the third application.

Case iv. A man, affected with stricture of the urethra for six years, called in M. Girard in consequence of a suppression of urine, which had lasted four days, notwithstanding the employment of general and local bleeding, bathing, anodynes, &c. The belladonna ointment was ordered: after the first friction a slight flow of urine took place, and the relief was complete after the continuance of the friction for thirty-six hours. *Journ. des Connaissances Méd.-Chir., Mai,* 1835.

II. *Employment of Belladonna in Spasmodic Contractions of the Uterus, Urethra, and Inguinal Ring.* By M. Carré, chief Surgeon of the Military Hospital of Briançon.

Case i. A lady was in labour of her third child; the waters had broke, and, as no progress was gained, the midwife attempted to dilate the os uteri by her fingers. This proceeding increased the irritation and contraction, and produced general convulsions. M. Carré, being called in, bled the patient and used the warm bath, but to no purpose. He then ordered the os tincæ to be rubbed with belladonna ointment every half-hour; and, after the third friction, the uterus became sufficiently dilated to permit the operation of turning, and the child was delivered, and lived. The ointment was made by rubbing up eight grammes of Ext. Belladonnæ with sixty-four grammes of cerate, and of this from two to four grammes were used each time.

Case ii. A woman, æt. twenty-one, was prematurely taken in labour at the eighth month. The waters had broken for some time, and, when M. C. was called, he found the os uteri so strongly contracted upon an arm of the fœtus, that he could not introduce his hand. Having first had recourse to bleeding, &c., the same ointment was applied, and, after the fourth friction, the dilatation was sufficient to permit the operation of turning, and the extraction of a dead child.

Case iii. A man had suffered from retention of urine for twenty-four hours, without any relief from bleeding and baths. The catheter could not be introduced beyond two inches, on account of the spasmodic contraction of the urethra. As the patient had been able to make water freely previously to the attack, M. C., believing the case merely spasmodic, prescribed the belladonna, which he applied by friction with the ointment on the glans, and by applying to the perineum a poultice made with decoction of the leaves, and further moistened with a solution of the extract. In an hour or two the urine began to flow slowly, and he was completely relieved in three hours.

Case iv. A man suffered a protrusion of voluminous inguinal hernia in attempting to lift a load. After ineffectual attempts at reduction, the use of bleeding, baths, &c., M. C. had recourse to M. Guerin's practice, introducing into the urethra a bougie covered with equal parts of cerate and extract of belladonna, and in half an hour's time he was able to reduce the hernia.

Case v. A soldier, subject for some years to a hernia, and for which he used a truss, had the misfortune to break this in leaping a ditch, and his hernia protruded and became strangulated. He had been ill twenty-four hours, suffering great pain, vomiting, &c., when M. C. saw him. Bleeding, baths, and the taxis were tried in vain. The tumour was then rubbed with the belladonna ointment, and a cataplasm applied. The pains ceased in from half an hour to three quarters, and, the taxis being then admissible, it proved readily successful.

CASE VI. Another soldier suffered in the same way, and was relieved by the belladonna ointment after the failure of other means.

*Journ. des Connaissances Méd.-Chir., Mai,* 1835.

III. *Two Cases of Incarcerated Hernia cured by the use of Belladonna Ointment.* By PIETRO PORTA, M.D. of San Zenone.

CASE I. A stout healthy man, æt. 50, upon lifting a heavy weight, was seized with a sudden pain, attended with tumour in the right iliac region. A medical man having recognized a crural hernia, bled the patient, and prescribed warm fomentations. The next day Signor Porta was called in, when the intense pain, meteorism, hiccough, vomiting, and obstipation, unrelieved by a second bleeding and the taxis, determined him to resort to the use of the belladonna, in the form of dried leaves ʒj. to lard ʒvj. This however could not be procured for a whole day, during which delay all the symptoms became much aggravated: nevertheless a few frictions with the ointment over the tumour caused it to disappear, with all its attendant symptoms.

CASE II. This was supposed to be a case of omental inguinal hernia, and occurred in a child of five years old. The tumour was inelastic, doughy, and irregular, giving rise to no prominent symptoms of suffering, but still, after several days, remaining irreducible by the taxis and warm baths. The belladonna ointment, applied every two hours for three days, succeeded in effecting the reduction, after the failure of every other means.

[Although the majority of the foregoing cases are far from presenting positive evidence of the efficacy of belladonna as a relaxer of the spasm present or presumed to be present in them, since similar cases terminating in like manner, without the use of this remedy, must have occurred to most surgeons of experience,—still they cannot be repudiated as unworthy the notice of the practitioner, according to the law of evidence commonly received in physic. To remove all doubt, a much greater number of successful cases must be adduced, or an equal number of similar cases must be treated with and without belladonna, and the majority of favorable results proved to be on the side of the treatment with this remedy. In respect of *hernia,* we must strongly protest against the adoption of any measures attended with loss of time and delay of the surgical operation, in a complaint of so urgent a nature as incarcerated hernia. When however, as sometimes occurs, through the strong opposition of the patient or his friends, an operation is impracticable, no mode of treatment which offers a chance of success should be neglected; and in such cases frictions with belladonna, harmless in themselves and soothing to the patient, are not only admissible, but are to be recommended, as supported by experience at least, if not by sound pathology.

DR. MOTARD, of Turin, has found that a solution of belladonna, introduced into the nose, dilates the pupil effectually; and he is in the habit of moistening a pinch of snuff with a solution, by which means the pupil next to the nostril in which it is introduced is dilated in a minute or two. The dilatation lasts about two days.—This hint is worthy of trial in those cases of cataract where the patients are in the constant habit of using belladonna to improve in some degree their imperfect vision; as it is a more convenient process than the common one.]

*Giornale delle Scienze Medico-Chirurgiche,* No. X. *Aprile,* 1835.

---

*On the Influence of Atmospheric Heat in the Cure of Wounds and Ulcers.* By M. JULES GUYOT, D.M.P.

[IT is not necessary for us to make any remarks upon this memoir. Some of the facts that it contains are interesting, and their bearing upon surgical practice, not to mention other considerations, is sufficient to recommend them to the attention of other experimenters and to practitioners.]

Rabbits were selected as the subjects of experiment, on account of the delicacy of their organization, and the facility with which their wounds suppurate. Three different kinds of apparatus were employed: the first consisted of a wooden box, divided into four compartments, one above the other, and each capable of containing two rabbits. These open behind by a slide, and in the front is a trough which contains the animal's food. The whole is traversed by a vertical tube, an aperture in which opens into each compartment, the size of which may be varied at will. Beneath the inferior extremity of this tube is placed a lamp, which burns constantly, and, the heated air passing through the tube, elevates every compartment to the requisite temperature. The degree of heat is ascertained by a thermometer passed into each compartment through an aperture in its side. A strong cloth is nailed over the front of this apparatus, with openings allowing the animals' heads to pass through them; so that the bodies of the rabbits remain in the heated air, whilst their heads are free in the external air, and they are able to eat from their troughs.

[As the experiments from which results of a practical character were derived were those made in this apparatus, it is unnecessary to describe the other two; one of which was for immersing an animal entirely in an uniformly elevated temperature, the other for the application of local heat.]

In the apparatus described rabbits can support, for days or weeks, a constant heat of from 113° to 147° Fahr., without sweat, without loss of appetite, or other disturbance than slight acceleration of respiration, which is not always observed, and which often ceases entirely. The same effects take place when the temperature is between 77° and 97° Fahr.; but without exception, when exposed to a heat between 97° and 113° Fahr., there has been extreme langour, with no appetite, and hurried respiration.

The following effects of heat thus applied on wounds have been observed:—Of four simple incisions, exposed constantly to a temperature of 140° Fahr., two united by the first intention, in from four to six hours; the other two remained gaping, and furnished during twelve hours a serous exudation, which gradually became dry, and formed over the surface of the wound a shining, rose-coloured, thin, transparent varnish, which gradually cracked, whilst the wound diminished in extent. On the fourth day cicatrization was complete. This process had apparently taken place without inflammation; no suppuration occurred. Two incisions exposed to 122°, and two to 158° Fahr., united in a few hours, with the same phænomena as those just mentioned. Two incised wounds, exposed to a temperature of 86° Fahr., healed less readily; and it was only after the seventh day that cicatrization, which occurred without either inflammation or suppuration, appeared sufficiently solid to be exposed to the open air: one of these cicatrices continued sound; the other, in the course of twenty-four hours, had ulcerated and began to suppurate. Two incised wounds, exposed to the open air at 57° Fahr., remained twenty-four hours without any apparent change; the borders of the wounds then swelled, and their surfaces furnished a considerable quantity of serosity. They afterwards became covered by greyish yellow, soft, and opaque crusts. Towards the fifth day pus could be detected beneath, and on the sixth it escaped; the incrustations were then taken off, when the wounds presented a red and healthy appearance. On the seventh day a new crust was formed, and the wounds had contracted. The pus again collected, on the eighth day, beneath the crusts, which were again removed. On the ninth and tenth days, the borders of the wounds approached each other, and were covered with a linear incrustation, which fell on the twelfth day, leaving perfect cicatrices.

Elevated temperature was next applied to wounds in various conditions,—some nearly healed, others half incrusted; some suppurating; and four quite sanious, pale, and making no progress towards cicatrization. In these various states they were placed in the apparatus already described; two at 104°, and one at 122° Fahr. Three other wounds were renewed, either by removing their crusts or by making new incisions; one was placed in a heat of 86°, the other of 140° Fahr. Two more were similarly renewed, and in two other rabbits fresh wounds were made in the muscular substance of the thighs; after which they were all exposed to elevated heat. On the following day, the suppuration of one wound had much diminished; the four sanious wounds were red and almost dry; those which were renewed were covered by the varnish; the two new wounds still furnished a serous exudation. On the second day, the suppurating and four sanious wounds had incrusted. The rose-coloured varnish was found upon the two fresh wounds, and over those which had been cleansed it remained without any suppuration during four days. During the night of the sixth day the lamp was extinguished. On the seventh, the thermometer being at 60° Fahr., the two cleansed and the two newly inflicted wounds were suppurating abundantly: the lamp was again lighted, the crusts removed, and the wounds cleansed. Three days were required to restore them to their previous state. By the fourteenth day of the immersion in the heated air no wounds remained uncicatrized.

At the suggestion of M. Magendie, M. Guyot was induced to try the effects of elevated temperature on the process of ulceration in man.

The apparatus employed is a box of an oblong shape, twelve inches long, and ten inches square at either end; closed at both extremities by a linen cloth nailed to the box, and having in it an aperture, through which the limb which is the subject of experiment may pass. This aperture is bordered by a running string, which allows of its being accurately fitted to the limb. The state of the wound is examined by a little door in the top of the apparatus. A tube is inserted into the bottom of the box horizontally, bent at a right angle; beneath its other extremity is a lamp. The degree of heat is regulated by a slide in the box, and estimated by a thermometer inserted into the same side. Those parts of the limb which are not contained in the box are supported by cushions; the whole is then fastened by tapes to the sides of the bed. The patient must observe perfect quietude.

Among the ulcers submitted to this mode of treatment was one of four years' duration, the sequel of a comminuted fracture, and which had resisted all the methods of treatment which had been employed; another, in an aged subject, old, painful, and indolent, which had occasionally cicatrized, but only for a short time; a third, of many years' duration, sanious, and covered with dark livid spots.

The effect of dry heat was favorable in all; in some it was continued until cure was effected. The ulcers became dry, inflammation and suppuration diminishing; pain was lessened, or entirely ceased; incrustation took place; the cicatrices were well formed, and shewed no tendency to ulcerate.

The previous observations having been submitted to the Academy of Sciences, M. Roux was appointed to verify them by more experiments.

[We may select the following case from those which are recorded, as most strongly illustrating the effects of heat upon wounds.]

An ulcer of eight years' duration, occupying the whole of the posterior, inferior, and external part of the left leg, having an average diameter of four inches, was subjected to a temperature of 97° Fahr. The ulcer had been variously treated, and with some benefit, but had arrived at a state in which for three months it had made no favorable progress, under any means employed. The cicatrix surrounding it was thin and tense; its surface bled on the least touch, and the ulcer was situated over an enormous swelling of the fibula, and extended into a depression, which was owing to the loss of the tendo Achilles. The leg was deformed, and in such a condition that M. Roux conceived the only hope for the patient to consist in amputation of the limb. The constitution of the individual was extremely irritable, and he unwillingly submitted to the treatment. During the first two days suppuration was abundant, and the leg and foot swelled; on the third day they diminished, and some incrustation took place. During five weeks (the time that the treatment was continued,) the healing process went on, and at the end of this period the ulcer measured only ten lines by six. The cicatrization now stopped, and the wound bled on the slightest touch. The patient left the hospital, being unwilling to submit to amputation, which was proposed to him.

The following conclusions are drawn by M. Guyot from his experiments:

1. Wounds have always healed more rapidly in a temperature above 85° Fahr., without dressing, than with or without dressing in a lower temperature.

2. Some wounds have healed in a heated atmosphere, which have not done so in one of the ordinary temperature.

3. In the former, the majority of wounds have healed without inflammation or suppuration; in the latter, this has not been observed.

4. Wounds have ceased to suppurate when exposed to heat, and have undergone the same healing process as fresh wounds.

From the effects of heat on man, it may be remarked that

1. An ulcer will heal, without any other means than the local application of increased temperature.
2. Heated air may give rise to the formation of a large cicatrix, in forty-eight hours, over an old ulcer.
3. In all cases, it aids, and constitutes one of the most favorable conditions for cicatrization.
4. Instead of giving rise to inflammation, it may check that which exists.
5. It acts to a certain degree on an internal malady.
6. It may be borne at 115° Fahr. during several weeks, without giving rise to serious accidents.

The employment of heat as a remedial agent, M. Guyot considers as evidently indicated in a certain number of cases: for instance, of local heat in wounds, ulcers, strumous engorgements, rheumatic pains, and white swellings; of diffuse heat (as by the first apparatus described,) in sciatica, accidental amenorrhœa, paraplegia; of general heat, in amenorrhœa of young women, the scrofulous diathesis, and phthisis. With regard to its beneficial operation in the last-mentioned disease, it is asked whether it may not be inferred, from the marked improvement which has followed the exposure of ulcers on the external surface to increased temperature, whether a similar advantage might not be derived from its application to internal ulcers, through the medium of respiration?

*Archives génerales de Médecine,* 3me Serie, tome 8. *Juillet,* 1835.

---

*On the Treatment of White Swellings.* By M. Lisfranc.

M. Lisfranc defines a white swelling to be a chronic enlargement of a joint. He does not attempt, as Sir B. Brodie has done, to classify diseases of articulations according to the tissue which is primarily affected. Even when the extreme mobility of the joint proves that the ligaments are destroyed, or when, on bending the joint, a grating sound is heard as if two surfaces rubbed together, he does not consider amputation indispensable. The most dangerous white swelling is a tumour which gives on pressure the sensation of a spongy tissue, never acquires a very considerable size, and does not always give pain. It is formed (as is ascertained by dissection,) of a reddish substance, like erectile tissue, in which there are granulations analogous to pulmonary tubercles. Suppuration soon takes place in this disease, and sanious pus with portions of the erectile tissue escapes. If amputation is not consented to, M. Lisfranc applies moxas, to endeavour to destroy the anormal tissue by inflammation.

*Treatment of White Swellings.* If there is any visceral disease, either preceding the local affection or coming on during the cure, M. Lisfranc directs his attention to it, and does not attempt to cure the disease of the joint; for in such cases he has found that a diminution of the local affection was followed by an aggravation of the visceral disease, and that the cure of the latter relieved the former malady. Absolute rest of the limb is necessary, in the position which will be most convenient, should anchylosis take place. In hip disease, M. L. fixes the leg, to prevent luxation. As regards treatment, it is important to ascertain whether the *stage* is acute or chronic: not that there is acute inflammation of the joint, but rather the state in which there is increased heat of skin, and permanent or remittent pain, which may be called sub-inflammation. In this stage local bleeding is beneficial, regard being had to the strength of the patient and to the effect of depletion upon the constitution: thus, in scrofulous and debilitated subjects, from twelve to fifteen leeches should be applied, and, if the patient is strong, from forty to fifty. In general, the blood should be allowed to flow for two hours. If the pain and heat continue, twenty more should be applied the next day, or the day after that. If great debility is produced, poultices and tepid baths are had recourse to, or narcotic applications, if the pain is increased. When the powers are invigorated, leeches should be reapplied until the tumour goes into the complete chronic stage. This may be after six weeks, or even after as many months. The return of the subacute symptoms requires leeches. M. Lisfranc has employed calomel and opium so as to produce rapid salivation, but not with much success: in the chronic stage he has not been more fortunate, but he intends to make more experiments with this medicine. When the chronic stage is fully established, and has at least existed eight or ten days, M. Lisfranc employs excitants. He considers that a few leeches determine a flow of blood to the part, and he therefore applies from four to ten, and allows them to bleed half or three-quarters of an hour. In some cases there is a diminution of the tumour the next day: it may, however, be increased in size, but this is generally temporary. If after six or eight days there is no improvement, the leeches must be repeated; but if, after two or three applications, the symptoms do not yield, other means must be used. If there is any diminution, from five to ten leeches should be applied every eight or ten days.

Indiscriminate compression is bad; but, when the tumour is soft and œdematous, it may succeed. If there is any probability of its reproducing the inflammation, a simple roller should be first applied: subsequently, stronger compression should be made by means of cones of agaric, two inches in height, with their bases resting on the tumour, and the mass passing at least half an inch beyond the swelling in its whole circumference. These are to be fixed with a roller. This compression should be continued for three months after the tumour is apparently cured, gradually diminishing the pressure. Kneading the joint previously to compression is useful in obstinate cases.

The actual cautery is remarkably beneficial when the tumour is so chronic that no pain is produced on walking. Hydriodate of potash, rubbed in externally, is the form of iodine which M. Lisfranc likes, and he only employs it in very chronic cases. Douche baths of all sorts, blisters, moxas, and setons, are sometimes useful: their effects must be carefully watched. After the patient is cured, exercise should be taken very cautiously and gradually.

*Revue Médicale, Avril et Mai*, 1835.

---

*Chlorine Gas as an Injection for the Cure of Hydrocele.* By M. DECONDÉ.

Dr. DEBLOIS, of Tournay, was in the habit of performing the radical cure of hydrocele by injecting chlorine gas instead of red wine. His premature death prevented him from making known the plan; but M. Decondé, who has seen its advantages, has described it. The chlorine gas is contained in a bladder, to which is fixed a pipe and stop-cock adapted to the canula of the trochar, into which it is fixed after the fluid has been evacuated: the stop-cock is then turned, and the bladder pressed so as to force the gas into the tunica vaginalis. When this is distended, the pipe and bladder are removed, and the thumb placed over the mouth of the trochar, so as to prevent the escape of the gas for the space of two minutes; it is then allowed to escape, and two or three repetitions of the injection are made, which are sufficient for the cure. The advantages are—the simplicity of the apparatus, and the whole sac being equally distended and exposed to the contact of the gas, which is not the case with fluid injections, which always gravitate. The danger also which sometimes occurs from the fluid being forced into the cellular tissue of the scrotum is avoided. M. Decondé proposes that the same remedy should be used in the cure of other diseases characterized by the secretion of serum in various cysts.—*Bulletin Médical Belge. Janvier*, 1836.

---

*On Traumatic Ophthalmia.* By Dr. SCHINDLER.

[CONSIDERING every fact as important which serves to illustrate the effects of injuries of the eye, we have perused with much satisfaction this paper by Dr. Schindler, whose name is already favorably known to British oculists, from his little work on Chronic Iritis after Keratonyxis.]

1. *From Burns.* The author first directs our attention to burns of the eye, and especially to burns from gunpowder. He remarks, that singeing of the eyebrows and eyelashes, formation of blisters on the eyelids and their edges, more or less considerable vesications on the sclerotica and cornea, and grains of unignited powder fixed in the cornea, are the less serious effects of an explosion; while, in more severe cases, the destroying force manifests itself in laceration of the iris, separation of the iris from the choroid, laceration of the retina, or even destruction of the whole textures of the eyeball. Setting aside the severe cases in which the preservation of the organ is impossible, the bad consequences of burns of the eye are by no means in direct proportion to the extent of the injuries received. Considerable lacerations are sometimes followed by no permanent bad effects; while, in other cases, the mere dazzling of the eyes by the sudden flash of light, especially when the explosion takes place very close to the eyes, produces the most disastrous effects on the sight. Dr. Schindler is of opinion that, in the last-mentioned cases, the over-stimulation produces an erethismus of the retina, passing into chronic inflammation and ultimate blindness. The patient, in one case particularly referred to, saw perfectly immediately after the explosion, and congratulated himself that his eyes were safe; but a speedy irritability to light, degenerating into severe photophobia, accompanied with slight external ophthalmia and considerable lacrymation, was the forerunner of an amaurosis erethica of the most dangerous description.

2. *From slight Injuries.* It sometimes happens, after apparently trivial injuries of the eyeball, as a small cut of the cornea, a painful degree of pressure on the eye, the eye being struck by a bit of turf, or the like, that there immediately follows such a deprivation of sight, that the patient perceives objects only as if through a thick cloud, or that the eye even loses entirely its sensibility to light. There is no pain, and at first no appearance of inflammation. It is only after some days that a slight redness of the conjunctiva, and sometimes of the sclerotica, sets in. The pupil is black, but motionless, and either regularly or irregularly dilated. By and bye, the iris becomes inflamed, but the patient manifests no intolerance of light. Hypopium, if it occurs in such a case, may be removed; but the eye will, notwithstanding, always become atrophic, the cornea remaining quite clear, and only gradually shrinking. Mr. Lawrence refers such symptoms to laceration of the retina; but this opinion, grounded solely on the sudden loss of vision, does not appear sufficiently borne out. The nature of the injury scarcely admits, in some of the cases, of such an explanation, and seems to point rather to a paralysis of the ciliary nerves.

3. *From severe Injuries.* Another and a very different form of traumatic ophthalmia arises from similar, but much more severe, injuries of the eye. Dr. Schindler has seen it follow a penetrating wound of the cornea or a blow with the fist. The patient, immediately after the injury, sees quite well, has no pain, and the redness of the conjunctiva is inconsiderable. The pupil is black and circular, not distorted, but slow in its motions. After some time, iritis comes on with its usual symptoms: the pupil, however, is at first considerably dilated, it loses its natural colour, and even in a few days becomes of a fine sea-green, as in some cases of glaucoma. At the same time vision is extinguished. The cornea and the aqueous capsule do not in general suffer, so that the change behind the pupil is distinctly seen. Dr. Schindler considers it as impossible to decide whether that change depends on an alteration of the choroid pigment or of the vitreous humour. If, in the progress of the case, the cornea and its lining membrane should become nebulous, the change of colour in the pupil can be perceived only so indistinctly that the observer will be at a loss whether to attribute it to cataract or to exudation. He will recognize his mistake, however, as soon as the cornea again clears. The iritis, in such cases running a subacute course, is attended with but little pain, and seldom produces any puriform effusion. After some weeks, the contracted pupil is closed by exudation, and of course all further observation of the deep-seated textures of the eye is prevented. This form also of traumatic ophthalmia always ends in atrophy, although Dr. Schindler acknowledges he long flattered himself that in such cases he might have preserved at least the form of the eye.

[We have frequently observed this form of traumatic ophthalmia. With regard to the green colour which appears behind the pupil, and which bears so close a resemblance to glaucoma, it is plain that either glaucoma has been actually induced by the injury or subsequent inflammation, or a state similar in its effects to glaucoma. It is now well known that glaucoma is no opacity of the vitreous humour, nor reflection from an opaque retina, but that it depends chiefly on a change of colour in the posterior lamellæ of the lens, by which these, having assumed a reddish brown or deep amber hue, absorb the violet and blue rays of the light entering the eye, leaving the yellow and green rays but little affected; whence results the green appearance of the humours in that disease.* Various substances in nature present, as is familiarly known, a different colour, according as they are seen by reflection or by refraction;† and the glaucomatous lens is one of them. Seen in the eye, by reflected light, it is green; seen out of the eye, by refraction, it is of a deep amber or reddish brown. Now, in the traumatic ophthalmia in question, either the lens has undergone a similar change of colour, as in glaucoma, or behind the lens there exists an effusion or deposition, productive of the same optical effect.] *Zeitschrift für die Ophthalmologie,* B. v. H. 1. 1835.

---

*On Foreign Bodies adhering to the Cornea, and the Manner of removing them.*
By Dr. SCHINDLER.

[THE observations in this paper apply only to those very minute foreign bodies of a black colour, which are called in Germany *Stahl-funken* (steel-sparks,) and occasionally in this country *fires.*]

It is remarkable that these bodies are met with only on the cornea, sometimes just at its edge, but generally near its centre or on its lower half, and never over the sclerotica. Notwithstanding their general resemblance, however, they differ in different cases. Sometimes they are particles of iron which have been projected in an ignited state against the eye, when a person is striking fire with flint and steel, sharpening steel instruments, or the like. In this case the particle, when viewed with the microscope, is not, as Weissenborn declared, angular, and likely by its sharp points to injure the cornea, and wedge itself in it; but, on the contrary, smooth and round, lying more or less firmly in the little pit which it had formed for itself in the cornea, and, even when it has remained there for weeks, leaving no oxide behind it. On removing such bodies, we never find any sloughy shreds of cornea attached to them, nor any brown burned spot left on the cornea. In other cases, the foreign bodies in question consist of minute, unignited, metallic splinters, driven with force against the eye; as sometimes happens in the act of filing. Being sharp and angular, they remain firmly wedged in the cornea: their fine points, having become oxidized, are apt to break off when the bodies themselves are removed, and form a reddish brown stain of the cornea. Shreds of the corneal conjunctiva are not unfrequently found adhering to such particles after they are removed.

Almost as common as these are little black bodies, not of metallic origin, often vegetable; sometimes, as is discovered by examining them with the microscope, the germs of grasses, in other cases particles of coal. With the naked eye it is often impossible to distinguish between these and metallic particles.

[Dr. Schindler does not think the presence of such foreign bodies as above described so very productive of danger to the eye as some have done. We agree with him in the opinion that the eye is more frequently destroyed by the rude attempts of ignorant people to remove such bodies from the cornea, than by allowing them to remain. That they should always be removed is certain, and we believe the best instrument for the purpose is a small, bent, elastic, silver spatula. The operator raises the upper eyelid of the patient with his thumb, taking care not to touch the lower eyelid nor the cilia of either eyelid; he now tells the patient to look at him, and with the edge of the spatula the foreign body is in general easily unseated. This is not the method recommended by Dr. Schindler, who uses a

* Philosophical Magazine, for June, 1832, page 470.

† Experiments and Considerations touching Colours. By the Rev. Robert Boyle, Third Part. Exp. 10 and 11. London, 1670.

camel-hair pencil for the purpose; a safer instrument certainly than the extraction-knife, but which we should think in many cases insufficient to effect the object in view.] *Zeitschrift für die Ophthalmologie*, B. v. H. 1. 1835.

---

*On the Power of Nature in curing severe Diseases of the Eye.* By Dr. LORCH.

[WE pass over the introductory matter contained in this paper, and give an account of the cases only, in a condensed form.]

CASE I. *Closure of the Pupil, with subsequent Restoration of Vision.* This seems to have been originally a case of scrofulous ophthalmia: three ulcers of the cornea formed, followed by three protrusions of the iris. These degenerated into a staphyloma racemosum, the pupil closed, and vision was lost. Calomel and senega were administered, the latter being a remedy much spoken of by some German practitioners in affections of the cornea; blue ointment, with belladonna, was rubbed in round the eye; and, after salivation took place, the patient took camphor and opium. The redness and pain abated, but the pupil continued closed. After some weeks, during which Dr. Lorch did not see the patient, (a girl of thirteen,) she began to see, and could at length read with the bad eye. He scarcely believed it; but found, on examination, that the three prolapsed bits of iris had quite shrunk, that three leucomata had formed, and that between these there was a small motionless pupil, opposite to which the cornea was luckily clear. An engraving illustrates the appearance of the eye.

CASE II. *Dislocation of the Lens, with Recovery of Sight without Operation.* This is the case of an English boy, of seven years of age, residing at Geisenheim, in whom the left crystalline capsule burst in consequence of a fall on the back of his head. The pupil was expanded and fixed, and the lens changed its place on every motion of the head. At first it was transparent, and its edge was seen to glance like a diamond. When the patient leaned forward, the upper third of the lens fell into the anterior chamber, while its lower edge rested on the dilated pupil; when he bent the head back, the lens fell again into its natural place. In the former position the patient saw better. At length the lens grew opaque, and sight became proportionally diminished.

[Dr. Lorch considers this case to have been one of capsulo-lenticular cataract; but we must confess that the proofs that the capsule was in this stage opaque, or that it was afterwards absorbed, do not appear to us satisfactory.]

Dr. Lorch administered small doses of calomel and sulphate of quina, and rubbed in mercurial salve round the eye. He seems to have been contemplating extraction, when the lens sunk down into the posterior chamber, and was gradually absorbed. The upper half of the pupil cleared first, vision improved, and at length the patient was able to distinguish the letters of a book. What is remarkable, the right eye seemed to be becoming like the left, when the patient left Germany for England.

[We may remark, that cases of the kind here related by Dr. Lorch are sometimes regarded as examples of spontaneous dislocation or prolapsus of the lens, the patient not being able to recall to his recollection the receiving of any blow on the eye or head likely to have given rise to a rupture of the crystalline capsule. There also appears to be generally a degree of amaurosis attendant on such cases.]

*Zeitschrift für die Ophthalmologie*, B. v. H. 1. 1835.

## Figures and Tables

**Fig 1 f1:**
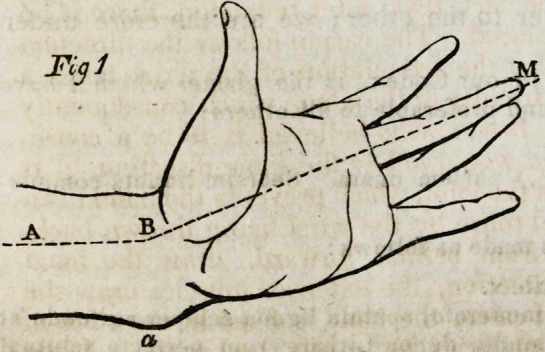


**Fig.2. f2:**
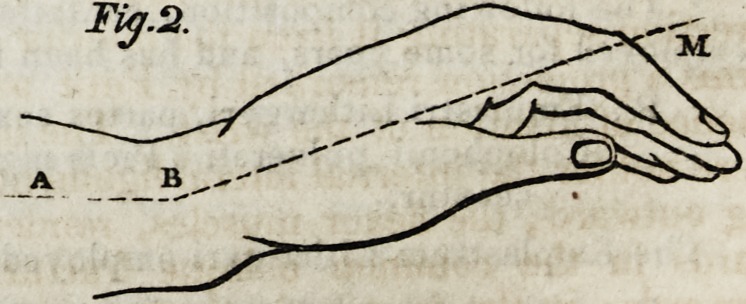


**Fig 3 f3:**
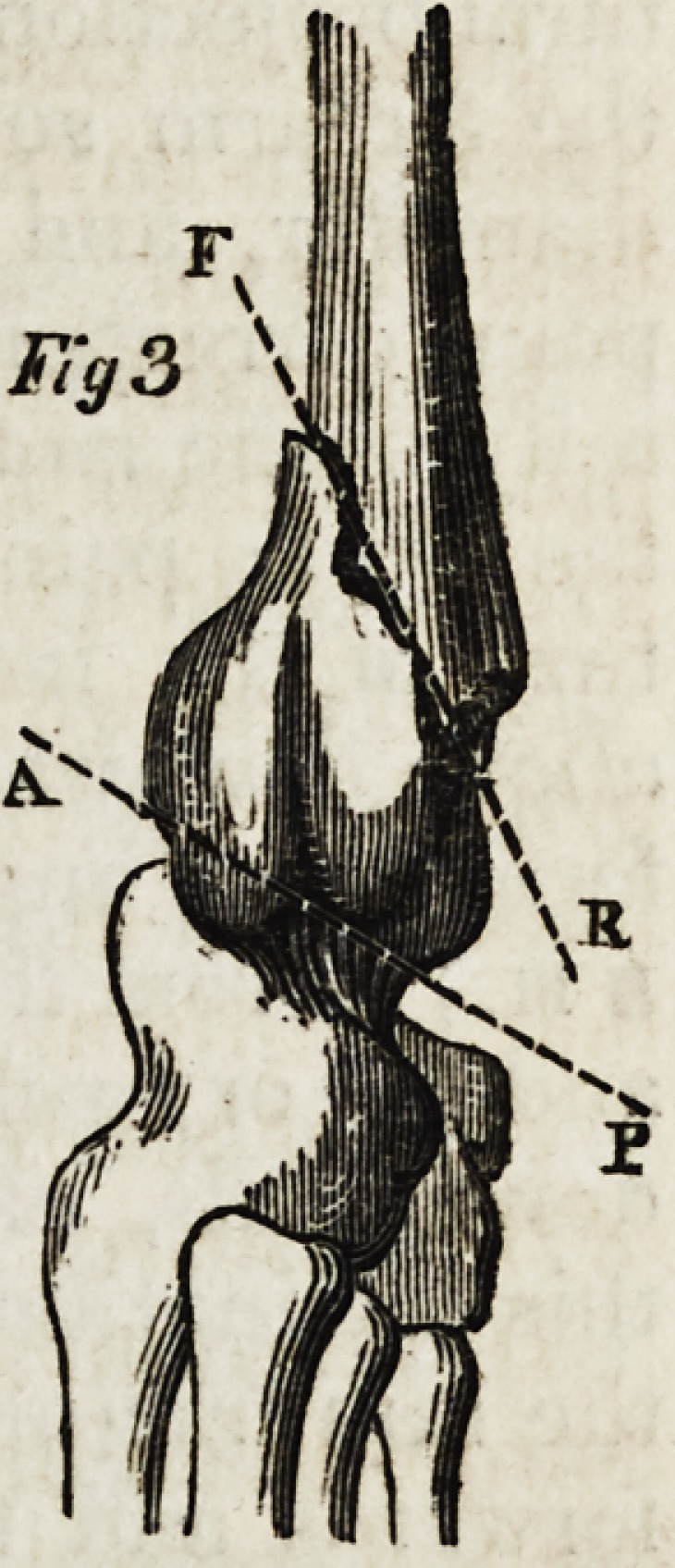


**Fig.4. f4:**
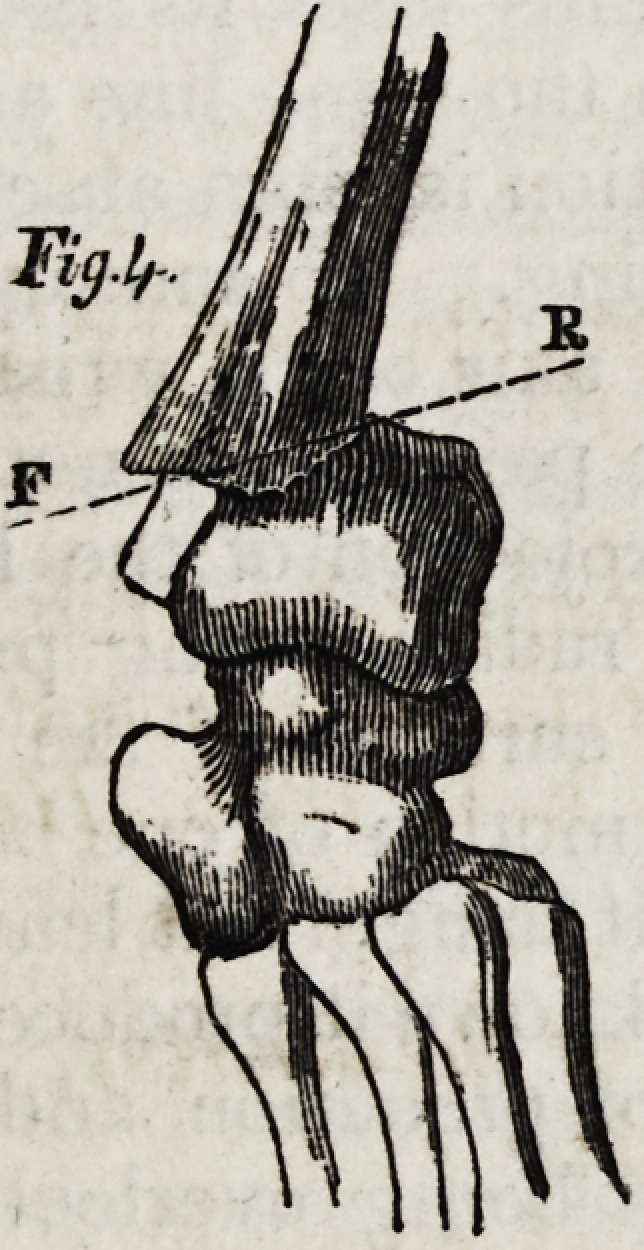


**Fig.5 f5:**